# Immunodominant Tuberculosis CD8 Antigens Preferentially Restricted by HLA-B

**DOI:** 10.1371/journal.ppat.0030127

**Published:** 2007-09-21

**Authors:** Deborah A Lewinsohn, Ervina Winata, Gwendolyn M Swarbrick, Katie E Tanner, Matthew S Cook, Megan D Null, Meghan E Cansler, Alessandro Sette, John Sidney, David M Lewinsohn

**Affiliations:** 1 Department of Pediatrics, Oregon Health and Sciences University, Portland, Oregon, United States of America; 2 Department of Molecular Microbiology and Immunology, Oregon Health and Sciences University, Portland, Oregon, United States of America; 3 Division of Pulmonary and Critical Care Medicine, Oregon Health and Sciences University, Portland, Oregon, United States of America; 4 Portland Veterans Administration Medical Center, Portland, Oregon, United States of America; 5 La Jolla Institute for Allergy and Immunology, San Diego, California, United States of America; Johns Hopkins School of Medicine, United States of America

## Abstract

CD8^+^ T cells are essential for host defense to intracellular bacterial pathogens such as Mycobacterium tuberculosis (Mtb), *Salmonella* species, and Listeria monocytogenes, yet the repertoire and dominance pattern of human CD8 antigens for these pathogens remains poorly characterized. Tuberculosis (TB), the disease caused by Mtb infection, remains one of the leading causes of infectious morbidity and mortality worldwide and is the most frequent opportunistic infection in individuals with HIV/AIDS. Therefore, we undertook this study to define immunodominant CD8 Mtb antigens. First, using IFN-γ ELISPOT and synthetic peptide arrays as a source of antigen, we measured *ex vivo* frequencies of CD8^+^ T cells recognizing known immunodominant CD4^+^ T cell antigens in persons with latent tuberculosis infection. In addition, limiting dilution was used to generate panels of Mtb-specific T cell clones. Using the peptide arrays, we identified the antigenic specificity of the majority of T cell clones, defining several new epitopes. In all cases, peptide representing the minimal epitope bound to the major histocompatibility complex (MHC)-restricting allele with high affinity, and in all but one case the restricting allele was an HLA-B allele. Furthermore, individuals from whom the T cell clone was isolated harbored high *ex vivo* frequency CD8^+^ T cell responses specific for the epitope, and in individuals tested, the epitope represented the single immunodominant response within the CD8 antigen. We conclude that Mtb-specific CD8^+^ T cells are found in high frequency in infected individuals and are restricted predominantly by HLA-B alleles, and that synthetic peptide arrays can be used to define epitope specificities without prior bias as to MHC binding affinity. These findings provide an improved understanding of immunodominance in humans and may contribute to a development of an effective TB vaccine and improved immunodiagnostics.

## Author Summary

CD8^+^ T cells are essential for host defense to intracellular bacterial pathogens such as Mycobacterium tuberculosis (Mtb), *Salmonella* species, and Listeria monocytogenes, yet little is known about the antigens recognized by human CD8^+^ T cells in response to tuberculosis (TB). TB, the disease caused by Mtb infection, remains one of the leading causes of illness and death worldwide and is a frequent complicating infection in individuals with HIV/AIDS. Therefore, we undertook this study to define commonly recognized CD8 Mtb antigens. First, we measured the frequencies of CD8^+^ T cells recognizing Mtb antigens known to be recognized by CD4^+^ T cell antigens in persons infected with Mtb. In addition, we identified the Mtb antigen and epitope recognized by several CD8^+^ T cell clones isolated from infected individuals. The epitope was presented to the T cell clones by an HLA-B allele in all but one case. We conclude that Mtb-specific CD8^+^ T cells are found in high frequency in infected individuals and are restricted predominantly by HLA-B alleles. These findings provide an improved understanding of how the human immune system recognizes intracellular pathogens and may contribute to the development of an effective TB vaccine and improved immunodiagnostics.

## Introduction

Infection with Mycobacterium tuberculosis (Mtb) remains an important cause of infectious disease, morbidity, and mortality worldwide [1] and has emerged as a major opportunistic infection in individuals with HIV/AIDS [2]. Control of infection with Mtb relies heavily on the cellular immune system, that is, the interaction of lymphocytes and Mtb-infected macrophages and dendritic cells (DCs) [3,4]. CD8^+^ T cells are associated with strong CD4^+^ T_H_1 cell responses, and are not only essential for effective immunity to viral pathogens, but also for immunity to some intracellular bacteria, such as Listeria monocytogenes and *Salmonella* species [5]. Increasing experimental evidence in the mouse tuberculosis (TB) model has suggested a protective role for CD8^+^ T cells in the host response. For example, adoptive transfer or in vivo depletion of CD8^+^ cells showed that this subset could confer protection against subsequent challenge [6–8]. β2-microglobulin–deficient mice, deficient in expression of major histocompatibility complex (MHC) class I, are more susceptible to Mtb [9] and to large doses of Bacille Calmette Guérin [10] infection than their wild-type littermates. This finding has been corroborated in CD8-deficient mice [11] and other mice deficient in class I processing and presentation [11–13]. However, mice lacking class Ia–restricted CD8^+^ T cells demonstrate more moderate susceptibility to Mtb infection [14,15]. In humans, Mtb-specific CD8^+^ T cells have been identified in Mtb-infected individuals and include CD8^+^ T cells that are classically, MHC-Ia, restricted [16–22], and non-classically, MHC-Ib, restricted by HLA-E [18,23], and by CD1 [24–26]. Taken together, studies of mice and humans support an important role for CD8^+^ T cells in TB immunity.

For most infections, the repertoire of the CD8 response is shaped by the entry of antigen into the MHC-I processing pathway, binding of peptides and/or non-peptide antigens to MHC-I molecules, and recognition of these structures by T cells. Ultimately, a relatively limited subset of pathogen-specific T cells emerge, a process that has been termed immunodominance [27]. While substantial effort has focused on defining immunodominant CD8 antigens for important human viral pathogens such as HIV and cytomegalovirus (CMV), little is known about the antigens recognized by human CD8^+^ T cell in response to intracellular bacterial infections. Furthermore, although a number of commonly recognized CD4 Mtb antigens have been described [28,29] (ESAT-6, CFP10, Ag85, etc.), surprisingly little is known about common Mtb antigens recognized by human CD8^+^ T cells. The majority of CD8^+^ epitopes that have been identified were defined by testing of Mtb peptides selected for high-affinity binding to MHC class Ia molecules (HLA-A2 in most cases; [19,20,30–34]). In almost all of these examples, however, the *ex vivo* frequency of these T cells in Mtb-infected individuals is low or undetectable, suggesting that these specificities may not represent immunodominant responses. In contrast, in the limited cases in which T cells have been used to define epitopes contained in selected Mtb antigens, high *ex vivo* frequencies have been demonstrated [17,35], suggesting that a T cell–centered approach can identify immunodominant epitopes. Moreover, CD8^+^ T cell responses to some Mtb antigens that represent good CD4 antigens (CFP10, ESAT-6, Ag85, and Mtb39) have been detected at high frequency in persons infected with Mtb [17–19,34]. Therefore, we used a limited library of overlapping synthetic peptides representing several known CD4 Mtb antigens to determine the magnitude of the CD8 response to these antigens in persons with active TB and latent TB infection (LTBI), as well as uninfected individuals. Furthermore, we utilized a panel of Mtb-specific CD8^+^ T cell clones to define minimal epitopes recognized within these antigens and determined the contribution of these novel epitopes to the *ex vivo* Mtb-specific CD8 response.

## Results

### Definition of Immunodominant Mtb-Specific CD8 Antigens

To define immunodominant Mtb-specific CD8 antigens, and to determine whether or not these responses result from infection with Mtb, we have used CD8^+^ T cells from uninfected donors, those with LTBI, or those actively infected with Mtb. Responses were determined either directly *ex vivo*, or using CD8^+^ T cell clones obtained by limiting dilution cloning on Mtb-infected autologous DCs [36]. As much is known about dominant CD4 Mtb antigens, a panel of these commonly recognized antigens was selected for further evaluation. These were Mtb39, CFP10, Mtb8.4, Mtb9.9A, ESAT-6, Ag85b, 19kDa, and EsxG. To avoid bias introduced by using peptides of predicted HLA binding specificity, we synthesized overlapping peptides (15 aa, overlap 11 aa) to represent the proteins of interest [17].

**Figure 1:**
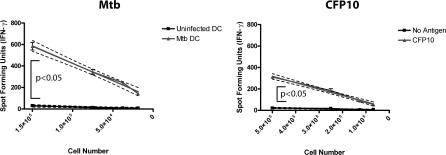
Determination of Human Effector Cell Frequencies *Ex Vivo* Using the IFN-γ ELISPOT Assay Magnetic bead–purified CD8^+^ T cells from a single donor (D466) were cultured with DCs (20,000/well) either infected with Mtb (H37Rv, multiplicity of infection = 50) or pulsed with peptide pool representing CFP10 (5 μg/ml each peptide; 15-mer overlap by 11 aa) in an IFN-γ ELISPOT assay. Each responding T cell population was tested in duplicate at four different cell concentrations. To determine the effector cell frequency of antigen-specific T cells, the average number of spot forming units per well for each duplicate was plotted against the number of responder cells per well. Linear regression analysis was used to determine the slope of the line, which represents the frequency of antigen-specific T cells. The assay was considered positive, reflecting the presence of a primed T cell response, if the binomial probability for the number of spots was significantly different by experimental and control assays, i.e., if the experimental line is statistically significantly different from the control line. The frequency of Mtb-specific and CFP10-specific T cells demonstrated in this figure was 1/307 and 1/1676, respectively.

To accurately determine the *ex vivo* effector cell frequencies of CD8^+^ T cells, linear regression analysis was used. As shown in Figure 1, using D466 as an example, magnetic bead–purified CD8^+^ T cells were tested against peptide-pulsed DCs over a range of CD8^+^ T cell numbers in an IFN-γ ELISPOT assay. A positive assay was determined as described below and if positive, the antigen-specific frequency was determined using linear regression.

**Figure 2:**
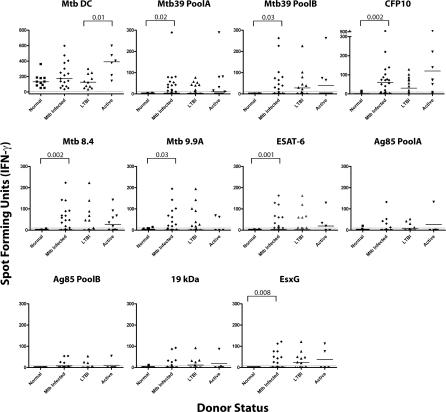
High Ex vivo CD8^+^ T Cell Frequencies to Mtb Antigens Are Associated with Mtb Infection As in Figure 1, to determine *ex vivo* CD8^+^ T cell frequencies, autologous DCs either infected with Mtb (graph labeled “Mtb DC”) or pulsed with cognate peptide pools representing known Mtb CD4 antigens (all other graphs) were incubated with CD8^+^ T cells in an IFN-γ ELISPOT assay. The number of spot forming units (SFU)/250,000 cells is shown, a number which is derived from linear regression analysis of the frequency of T cells tested at concentrations of 5 × 10^5^, 2.5 × 10^5^, 1.2 × 10^5^, and 6 × 10^4^ cells/well. Individuals without evidence for Mtb infection (“normal”); those with LTBI, and those with active TB (“active” defined as culture confirmed pulmonary TB) were evaluated. “Mtb Infected” includes both the “LTBI” and “active” groups. The number of individuals tested for each antigen were: Mtb infection (normal, *n* = 11; LTBI, *n* = 11; active, *n* = 6); Mtb39 Pool A (normal, *n* = 14; LTBI, *n* = 20; active, *n* = 10); Mtb39 Pool B (normal, *n* = 14; LTBI, *n* = 20; active, *n* = 10); CFP10 (normal, *n* = 14; LTBI, *n* = 20; active, *n* = 10); Mtb8.4 (normal, *n* = 14; LTBI, *n* = 20; active, *n* = 10); Mtb9.9A (normal, *n* = 14; LTBI, *n* = 20; active, *n* = 5); ESAT-6 (normal, *n* = 14; LTBI, *n* = 20; active, *n* = 5); Ag85 Pool A (normal, *n* = 14; LTBI, *n* = 17; active, *n* = 5); Ag85 Pool B (normal, *n* = 14; LTBI, *n* = 17; active, *n* = 5); 19kDa (normal, *n* = 12; LTBI, *n* = 17; active, *n* = 5); and EsxG (normal, *n* = 14; LTBI, *n* = 17; active, *n* = 5). *p*-Values are noted where statistically significant differences between groups are noted (*p* = <0.05; Wilcoxon/Kruskal–Wallis).

Uninfected individuals (*n* = 14), those with LTBI (*n* = 20), and those with active TB (*n* = 12) were evaluated for CD8 responses to a panel of Mtb CD4^+^ T cell antigens, as well as to Mtb-infected DCs. All individuals tested had robust CD8^+^ T cell responses to Mtb-infected DCs and were of greater magnitude in individuals with active TB than in those with LTBI (*p* = 0.01; Figure 2; Table 1). However, CD8^+^ T cell responses to the panel of Mtb antigens were found almost exclusively in those infected with Mtb in that statistically significant differences between uninfected and Mtb-infected individuals were noted for seven of ten antigens for both the magnitude of the response (Figure 2) and the proportion of positive assays (Table 1). However, differences in CD8^+^ T cell responses between individuals with active TB and LTBI were not statistically different. While strong CD8^+^ T cell responses were observed against many of the antigens tested, it is equally notable that several individuals with strong Mtb-directed CD8^+^ T cell responses did not have demonstrable responses to many of the antigens tested (unpublished data).

**Table 1:**
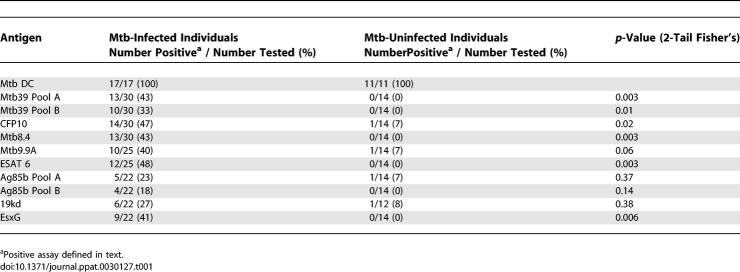
CD8^+^ T Cell Responses to Known TB Antigens

### Definition of the Minimal Epitope Recognized by Mtb-Specific CD8+ T Cell Clones

**Figure 3:**
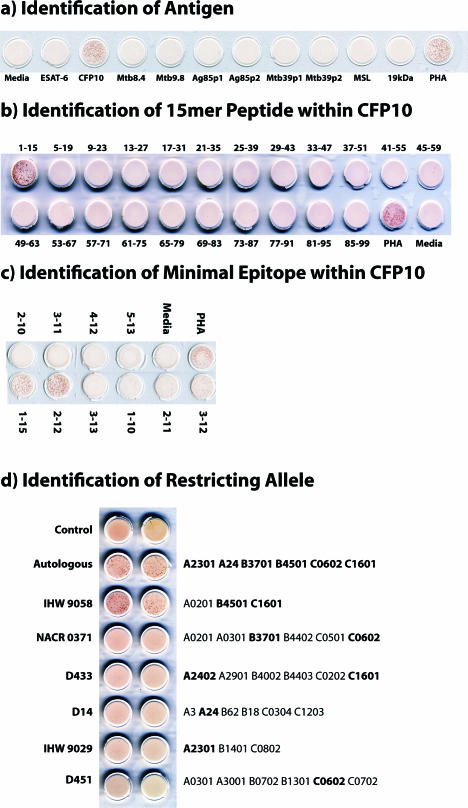
Definition of Antigenic Specificity and HLA Restriction: Characterization of T Cell Clone D466 D6 (A–C) To identify the antigen and minimal epitope recognized by T cell clone D466 D6, T cells (5,000 cells/well) were incubated with autologous LCL (20,000/well) and antigen (5 μg/ml). Negative controls (media, no antigen) and positive controls (phytohemagglutanin [PHA], final, 10 μg/ml) are shown for each assay. IFN-γ was assessed by ELISPOT after 18 h of co-culture. Pictures of ELISPOT wells are shown. (A) Antigens consisted of peptide pools representing known CD4 Mtb antigens, made up of 15-aa peptides overlapping by 11 aa. (B) Antigens consisted of individual 15-aa CFP10 peptides that together constitute the peptide pool. (C) Antigens consisted of individual nested peptides within CFP10_1–15_ (10 aa, 9 aa, or 8 aa), used to further map the epitope. (D) The restricting allele was identified using LCLs (20,000/well) that were either autologous or expressing HLA alleles matching D466 at one or two alleles (shown in bold font), pulsed with CFP10_2–10_ (5 μg/ml) as APC. Autologous LCLs without peptide (control) are also shown. After 2 h, cells were washed and incubated with T cells (500 cells/well) in an IFN- γ ELISPOT assay. Pictures of ELISPOT wells are shown.

**Figure 4:**
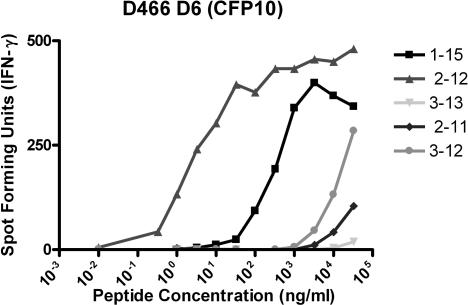
Confirmation of Minimal Epitope Mapping of D466 D6 To confirm the minimal epitope, autologous LCLs (20,000/well) were pulsed with peptide at the concentration indicated and co-cultured with T cells (1,000 cells/well). IFN-γ was assessed by ELISPOT after 18 h co-culture. Each point represents the mean of duplicate determinations.

**Figure 5:**
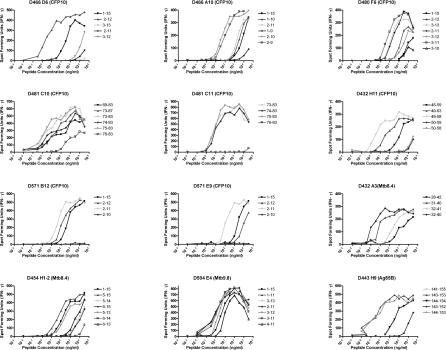
Summary of Minimal Epitope Mapping Data To determine the minimal epitope, autologous LCLs (20,000/well) were pulsed with peptide at the concentration indicated and co-cultured with T cells (1,000 cells/well). IFN-γ was assessed by ELISPOT after 18 h co-culture. Each point represents the mean of duplicate determinations.

These *ex vivo* frequency data demonstrated the presence of high-frequency responses to a number of known Mtb antigens, but did not shed light on the restricting allele, minimal epitope, or dominance hierarchy within the gene of interest. To address this question, we performed limiting dilution cloning of human CD8^+^ T cells using Mtb-infected DCs [36], and generated panels of both classically and non-classically HLA-restricted CD8^+^ T cell clones. Using peptide pools representing known CD4 antigens, the antigenic specificity of the HLA-Ia-restricted clones was defined in more than half of the clones (Table 2). This approach is demonstrated in detail for a single representative clone, D466 D6, derived from an individual with active TB. As shown in Figure 3A, testing the clone against autologous DCs pulsed with a panel of peptide pools unambiguously defined the antigenic specificity as CFP10. The clone was then tested against each of the 15-mer peptides that comprise the CFP10 pool, revealing that the epitope was contained within CFP10_1–15_ (Figure 3B). Each possible 8-aa, 9-aa, 10-aa, and 11-aa peptide was then synthesized and tested for reactivity, revealing antigenic activity between aa 2–11 (Figure 3C). Similarly, each clone was tested against lymphoblastoid cell lines (LCLs) sharing at least one HLA type with the donor (Figure 3D). Autologous LCL and IHW 9058 LCL, which share B4501 and C1601, presented the epitope to the clone, identifying both B4501 and C1601 as possible restricting alleles. However, C1601^+^ D433 LCL did not present the epitope, eliminating C1601 as a candidate-restricting allele. Therefore, D466 D6 was restricted by HLA-B4501. As demonstrated in Figure 4, by testing each plausible epitope over a broad range of concentrations, the minimal epitope was defined as CFP10_2–12_ for D466 D6. Experimental data supporting the assignment of the minimal epitope is provided for each clone in Figure 5, and a summary of the antigenic specificity, minimal epitope, and HLA-restricting allele is presented in Table 3. Unexpectedly, all but one of the T cell clones were restricted by HLA-B alleles. Furthermore, a minority of those observed were 9 aa in length.

**Table 2:**
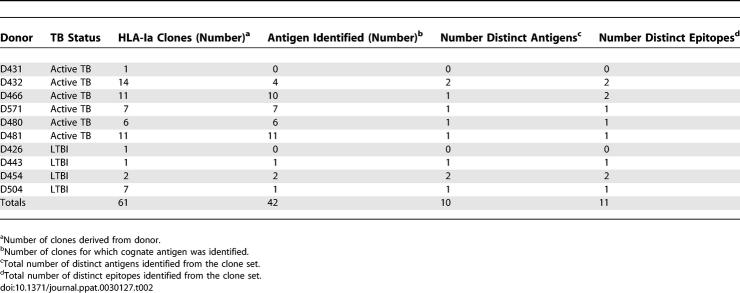
Many CD8^+^ T Cell Clones Recognize Known CD4 Antigens

**Table 3:**
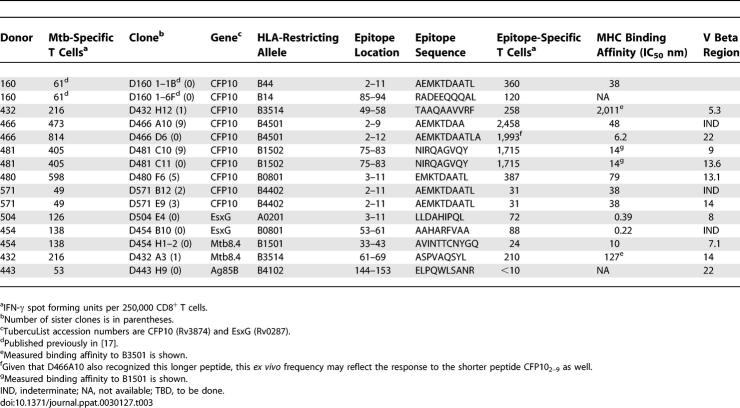
Summary of CD8+ T Cell Epitopes Identified

### Immunodominance of MTb CD8 Epitopes in Infected Individuals

**Figure 6:**
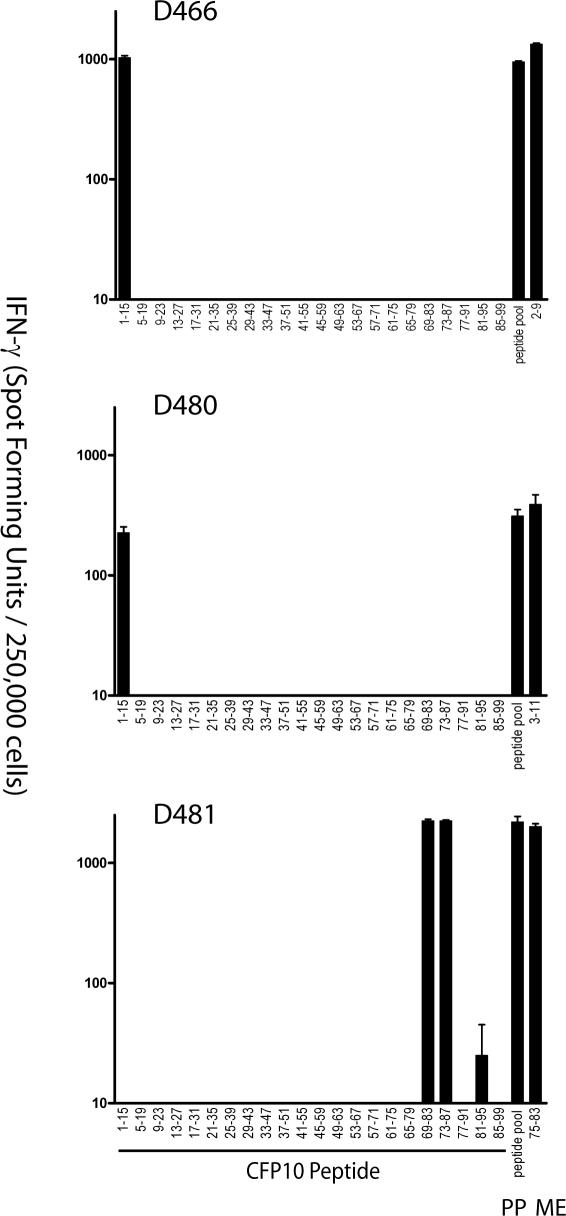
Profiling of Immunodominance Pattern for CFP10 To determine the effector cell frequencies, autologous DCs (20,000/well) were pulsed either with each individual 15-mer peptide (5 μg/ml), the peptide pool (PP; 5 μg/each peptide), or the minimal epitope (ME) determined from T cell clones derived from each donor (D466:CFP10_2–11_; D480:CFP10_3–11_; D481:CFP10_75–83_; 5 μg/ml), and tested against 250,000 magnetic bead–purified CD8^+^ T cells. IFN-γ release was assessed by ELISPOT after 18 h of co-culture. Each point represents the mean of duplicate determinations.

Because each of the individual CD8^+^ T cell clones was derived based on growth in the presence of Mtb- infected DCs, we sought to determine whether or not the antigen and epitopes identified reflected immunodominant epitopes *ex vivo*. Two independent approaches were pursued, the first to determine if the response was present at high frequency, and the second to determine what proportion of the total response to the antigen was constituted by the epitope. To determine the *ex vivo* effector cell frequency, as described in Figure 1, each epitope was tested using autologous DCs and magnetic bead–purified CD8^+^ T cells derived from the donor from whom the T cell clones was isolated. A summary of the *ex vivo* epitope-specific effector cell frequencies is presented in Table 3. For comparison, effector cell frequencies using autologous DCs infected with Mtb as the antigen-presenting cells (APCs) are shown as well. For 11 CD8^+^ T cell clones recognizing distinct epitopes, the epitope-specific frequency exceeded 50% of the total Mtb-specific CD8^+^ T cell response. For six of these clones, the epitope-specific frequency actually exceeded the total frequency of Mtb-specific CD8^+^ T cells. Conversely, for two clones, the epitope-specific T cell frequency constituted the minority of the total Mtb-specific CD8^+^ T cell response. Thus, overall, the epitopes reflected high-frequency responses, and could be considered a response that has been primed by exposure to Mtb. Notably, T cell clones isolated from four donors recognized CFP10. To determine if the epitopes defined reflected a substantial proportion of the total response to the antigen of interest, magnetic bead–purified CD8^+^ T cells from three donors with sufficient available peripheral blood mononuclear cells (PBMCs) were tested for reactivity to each individual 15-mer peptide, the peptide pool, and peptide representing the minimal epitope. As is demonstrated in Figure 6, the *ex vivo* frequencies to the minimal epitope, 15-mer peptide(s) containing the minimal epitope, and peptide pool were remarkably concordant. These data, then, suggest that for each donor a dominance hierarchy has been clearly established, and was reflected in the original clones. Finally, as is noted in Table 3, daughter clones of identical specificity were frequently identified, a result that would be predicted based on an immundominance hierarchy. TCR V beta staining was used to confirm the clonal relationship between daughter clones. Interestingly, in two cases, the identical minimal epitope and HLA restriction was represented by two distinct clones (Table 3).

### HLA Binding Affinity to CD8^+^ T Cell Epitopes

Because much work on human CD8^+^ T cell responses to Mtb has relied upon the use of HLA prediction algorithms, as each epitope was defined we asked whether or not the epitopes would have been predicted by these approaches. Given the prevalence of HLA-B alleles and 10-mer and 11-mer epitopes, it is perhaps not surprising that many of these epitopes were not ranked strongly (unpublished data). This left open the possibility that either HLA binding was indeed not predictive of antigenicity, or simply highlighted the limitations of those algorithms at the time they were used. To address this question experimentally, the IC_50_ for each peptide that had been synthesized in the course of definition of the minimal epitope was determined against a panel of human HLA molecules (Table S1). Shown in Table 3 is the IC_50_ for the minimal epitope with the cognate restricting allele. These data demonstrate that the T cell epitopes bound avidly to HLA, and show a high degree of concordance between the T cell epitope data and HLA binding data (Figure 5; Table S1).

## Discussion

Although the complete repertoire of CD8^+^ T cell responses in Mtb remains incompletely characterized, the following conclusions can be drawn. First, CD8^+^ T cell responses are present in persons infected with Mtb at frequencies that are comparable to that seen following many common viral infections such as vaccinia, influenza, and CMV [37,38]. This conclusion is based both on the pooled peptide experiments described above, and on the observation that when defined, dominant epitopes are present at high *ex vivo* frequencies. Conversely, we have not observed high-frequency responses to CD4^+^ T cell antigens in those without evidence of infection with Mtb. This observation strongly supports the hypothesis that these responses reflect an adaptively acquired response to infection with Mtb, rather than an innate response. By contrast, CD8^+^ T cell responses to Mtb-infected DCs were equivalent in infected and uninfected individuals. Using limiting dilution analysis, we have studied the proportions of Ia- versus Ib- restricted CD8^+^ T cells in Mtb-infected compared to uninfected individuals and have noted that Ib-restricted CD8^+^ T cell responses predominate in uninfected individuals (D. Lewinsohn, unpublished data). Therefore, we speculate that the response detected to Mtb-infected DCs in uninfected individuals may reflect chiefly a Ib-restricted response. While we did not observe an association of responses to specific antigens with disease status, a larger study might reveal more subtle differences.

All but one of the epitopes that have been mapped to date are restricted by HLA-B molecules. The reasons for this possible skewing are not yet clear. It is a formal possibility that the cloning methods we used biased isolation of HLA-B- over HLA-A-restricted T cell clones. However, using identical cloning methodology, we have isolated T cell clones specific for vaccinia, CMV, and influenza that did not display a preference for HLA-B restriction (D. Lewinsohn, unpublished data). Furthermore, in all three cases where we tested *ex vivo* the entire set of peptides representing CFP10, the entire response mapped to the HLA-B-restricted epitope (Figure 6). Therefore, we speculate that Mtb antigens may preferentially bind to HLA-B molecules, that Mtb preferentially interferes with HLA-A processing and presentation, that infection with Mtb leads to selective upregulation of HLA-B, or that HLA-B is preferentially delivered to the Mtb phagosome. Nonetheless, these data are consistent with those reported by Kiepiela et al. [39], in which HIV-specific T cell responses were found to be 2.5-fold more likely to be HLA-B- than HLA-A-restricted, and observed that viral load was more closely linked to HLA-B than HLA-A alleles. Human infection with mycobacteria has been demonstrated in pre-urban Egypt [40] and in pre-Columbian Peru [41], observations consistent with the hypothesis that mycobacterial infection has driven the diversity and peptide-binding repertoire of the HLA-B locus. Furthermore, our data is consistent with the hypothesis that the diversity of HLA-B alleles is related to the containment of both viral and bacterial pathogens. Hence, delineation of immunodominant epitopes and antigens within the context of important human pathogens will likely require careful evaluation of those epitopes presented by HLA-B.

While the immune response within an individual to a given antigen is narrowly focused, dominant epitopes that would be useful for population-based analysis have yet to be defined. This conclusion is based on the fact that few of the HLA-A2 epitopes described to date have proved to be widely recognized, and, most importantly, on the observation that a wide variety of HLA alleles appear to be used in the recognition of Mtb antigens. The antigen CFP10 is an excellent case in point. As is demonstrated in Table 3, T cell clones have been used to define high-frequency epitopes restricted by a variety of HLA alleles. While the N-terminal 15 aa could reasonably be considered immunogenic, in all but one case (CFP10_2–11_; HLA-B44) the minimal epitope defined has been unique to the individual from whom the T cells were derived.

By using a T cell–driven approach to epitope identification, it is possible to define dominant epitopes in humans infected with Mtb. While it is striking that for several of the T cell clones the *ex vivo* frequency of epitope-specific T cells was equal to or exceeded the total *ex vivo* frequency of Mtb-specific T cells, we acknowledge that the use of peptide-loaded DCs to determine T cell frequency likely overrepresents the proportion of the total Mtb-specific CD8^+^ T cell responses. Possible reasons are that Mtb infection is likely to produce relatively less cognate peptide compared to peptide loading, and that Mtb infection could possibly interfere with class I processing and presentation. Although the current observations are limited to a small panel of known CD4 antigens, current work is underway to perform a genome-wide survey of dominant CD8 antigens in Mtb. Definition of this panel will likely prove useful in the further study of the natural history of infection with Mtb and in the design of novel vaccines and diagnostics.

In TB, evaluation of epitopes based strictly on HLA binding algorithms has focused on HLA-A2, and has often failed to define dominant epitopes. Our observation that many of the epitopes are HLA-B restricted, and often longer than 9 aa, for which these algorithms are less robust, may help explain these findings. However, when evaluated experimentally, all of the minimal epitopes exhibit high-affinity binding to the cognate-restricting allele. In this regard, substantial progress in HLA prediction algorithms is evident [42,43], and could facilitate more efficient identification of dominant epitopes. Here, more information with regard to longer peptides will be of benefit. Finally, we speculate that these caveats are likely to apply to other intracellular pathogens as well.

One limitation to current knowledge is that the responses in humans have been made at a single time point. In this regard, a feature of Mtb is the chronic exposure to antigen that may persist for many years. How this chronic infection influences the shaping of the immune response and dominance repertoire is an important question that remains unresolved. The advent of new reagents for immunologic studies in the mouse model [44] will likely be useful in this regard. For example, chronic antigenic exposure seems likely to alter the affinity of the T cell response over time. Furthermore, it is possible that such long-term infection might lead to clonal exhaustion or dysfunction as has been described for chronic viral infection [38]. Finally, given the very high-frequency responses that we and others have observed to Mtb-infected DCs and to single antigens, it appears that the immune response to Mtb occupies a sizable fraction of the host's immunological activity, similar to that previously observed for infection with CMV [45]. If so, then this may have important implications for the aging immune system, and potentially for the requirements for the long-term containment of intracellular infection. For example, it is possible that threshold Mtb-specific CD8 frequencies are necessary for the maintenance of a state of chronic persistence. Conversely, the substantial immunological effort directed at Mtb may limit the host's ability to effectively combat novel infections. As a result, this static picture leaves open important questions as to the evolution of the Mtb-specific responses, and its relationship with chronic infection with Mtb.

It seems likely that Mtb has evolved potent mechanisms to modulate the immune response. At present, specific mechanisms for MHC-I immune modulation have not been described. However, it appears that TLR II stimulation via the Mtb-derived 19-kDa lipoprotein can modulate both MHC-I and MHC-II antigen processing, and can interfere with IFN-γ signaling [46–49]. Further work on the T cell subsets important in Mtb, including the immunodominant epitopes, will extend our understanding of the immunology of TB and potentially contribute to the development of a vaccine against this major killer.

How the Mtb-specific CD8^+^ T cell response fits into the natural history of infection with Mtb remains poorly characterized. For example, the ontogeny of the CD8^+^ T cell response relative to infection with Mtb remains unknown, as does the relationship of CD8^+^ T cell frequencies with regard to bacterial burden. However, by defining commonly recognized CD8^+^ T cell antigens and epitopes, it will become increasingly possible to employ direct *ex vivo* analysis to more precisely define Mtb-specific T cell responses in various subject groups of particular interest.

## Materials and Methods

### Study participants.

Study participants, protocols, and consent forms were approved by the Oregon Health and Science University institutional review board. Informed consent was obtained from all participants. Uninfected individuals and individuals with LTBI were recruited from employees at Oregon Health and Science University as previously described [36]. Uninfected individuals were defined as healthy individuals with a negative tuberculin skin test and no known risk factors for infection with Mtb. Individuals with LTBI were defined as healthy persons with a positive tuberculin skin test and no symptoms and signs of active TB. Individuals with active TB were recruited via institutional review board–approved advertisement and were self-referred from the Multnomah County TB Clinic, Portland, Oregon, United States, or from the Washington County TB Clinic, Hillsboro, Oregon, United States. In all active TB cases, pulmonary TB was diagnosed by the TB Controller of these counties and confirmed by positive sputum culture for Mtb. PBMCs were isolated from whole blood obtained by venipuncture or apheresis.

### Media and reagents.

Culture medium consisted of RPMI 1640 supplemented with 10% fetal bovine sera (BioWhittaker, http://www.cambrex.com/), 5 × 10^−5^ M 2 ME (Sigma-Aldrich, http://www.sigmaaldrich.com/), and 2 mM glutamine (GIBCO BRL, http://www.invitrogen.com/). For the growth and assay of Mtb-reactive T cell clones, RPMI 1640 was supplemented with 10% human serum. Mtb strain H37Rv was obtained from the American Type Culture Collection (http://www.atcc.org/) and prepared as previously described [36]. Peptides were synthesized by Genemed Synthesis (http://www.genemedsyn.com/). Synthetic peptide pools consisted of 15 mers overlapping by 11 aa, representing Mtb proteins demonstrated to be potent CD4 antigens. Peptide pools representing CFP10 [50,51], ESAT-6 [52], Mtb39a (two pools, A & B) [53], Mtb8.4 [54], Mtb 9.9A [16], EsxG [55,56], 19kDa antigen [57], and antigen 85b (two pools, A & B) [58] were synthesized. Peptides were resuspended in DMSO, and up to 50 peptides were combined into one pool such that each peptide in the pool was at a concentration of 1 mg/ml. Peptide pools were stored at −80 °C.

### Cell lines and T cell clones.

EBV-transformed B cell lines, LCLs, were either generated in our laboratory using supernatants from the cell line 9B5–8 (American Type Culture Collection) or obtained from the National Marrow Donor Program (http://www.marrow.org/). LCLs were maintained by continuous passage as previously described [18]. Mtb-specific T cell clones were isolated from individuals with LTBI or active TB, using Mtb-infected DCs as APCs and limiting dilution cloning methodology as previously described [36]. Briefly, CD8^+^ T cells were isolated from PBMCs using negative selection using CD4 antibody-coated beads and then positive selection using CD8 antibody-coated magnetic beads per the manufacturer's instructions (Miltenyi Biotec, http://www.miltenyibiotec.com/) or via flow cytometry. In this case, CD4-PE (BD Biosciences, catalog #555347; http://www.bdbiosciences.com/) negative and CD8-APC (BD Biosciences, catalog #555369) positive cells (purity >99%) were sorted on a Becton Dickinson LSR II (http://www.bd.com/). T cells were seeded at various concentrations in the presence of a 1 × 10^5^–irradiated autologous Mtb-infected DC, generated as described below, and rIL-2 (5 ng/ml) in cell culture media consisting of 200 μl of RPMI 1640 supplemented with 10% human sera. Wells exhibiting growth between 10–14 d were assessed for Mtb specificity using ELISPOT and Mtb-infected DCs as a source of APCs. T cells retaining Mtb specificity were further phenotyped for αβ T cell receptor expression and CD8 expression by FACS and expanded as described below. V beta usage was determined using the IOTest Beta Mark Kit from Beckman Coulter, catalog #IM3497 (http://www.beckmancoulter.com/).

### Expansion of T cell clones.

To expand the CD8^+^ T cell clones, a rapid expansion protocol using anti-CD3 mAb stimulation was used as described previously [18].

### Generation and infection of peripheral blood DCs.

Monocyte-derived DCs were prepared according to a modified method of Romani et al. [18,59]. To generate Mtb-infected DCs, cells (1 × 10^6^) were cultured overnight in the presence of Mtb at a multiplicity of infection = 50:1. We have determined that this multiplicity of infection is optimal for detection of Mtb-specific CD8^+^ T cells, as heavy infection is required to optimize entry of antigen into the class I processing pathway [60]. After 18 h, the cells were harvested and resuspended in RPMI/10% human serum.

### MHC binding assays.

The MHC peptide binding assay utilized measures the ability of peptide ligands to inhibit the binding of a radiolabeled peptide to purified MHC molecules, and has been described in detail elsewhere [61] . Briefly, purified MHC molecules, test peptides, and a radiolabeled probe peptide are incubated at room temperature in the presence of human β2-microglobulin and a cocktail of protease inhibitors. After a 2-d incubation, binding of the radiolabeled peptide to the corresponding MHC class I molecule is determined by capturing MHC/peptide complexes on W6/32 antibody (anti-HLA A, B, and C) or B123.2 (anti-HLA B, C, and some A) coated plates, and measuring bound cpm using a microscintillation counter. For competition assays, the concentration of peptide yielding 50% inhibition of the binding of the radiolabeled peptide is calculated. Peptides are typically tested at six different concentrations covering a 100,000-fold dose range, and in three or more independent assays. Under the conditions utilized, where [label] < [MHC] and IC_50_ ≥ [MHC], the measured IC_50_ values are reasonable approximations of the true Kd values.

### IFN-γ ELISPOT assay.

The IFN-γ ELISPOT assay was performed as described previously [18]. For determination of *ex vivo* frequencies of CD8^+^ T cells responding to Mtb infection or Mtb antigens, CD8^+^ T cells were positively selected from PBMCs using magnetic beads (Miltenyi Biotec) such that >97% of the cell population were CD8^+^ T cells. These CD8^+^ T cells were used as a source of responder T cells and tested in duplicate at four different cell concentrations (5 × 10^5^, 2.5 × 10^5^, 1.2 × 10^5^, and 6 × 10^4^ cells/well). Autologous DCs (20,000 cells/well) were used as APCs, and DCs were either infected with Mtb or pulsed with peptide pools (5 μg/ml, final concentration of each peptide) and then added to the assay. For assays using T cell clones, T cells (1,000 or 5,000 cells/well) were incubated with autologous LCL (20,000 cells/well) in the presence or absence of antigen. Negative and positive controls were included in each assay and consisted of wells containing T cells and DCs either without antigen or without antigen but with inclusion of phytohemagglutanin (PHA, 10 μg/ml; EMD Biosciences, http://www.emdbiosciences.com/), respectively. For all assays, responding T cells were incubated with APCs overnight.

### Data analysis.

To determine the *ex vivo* frequency of antigen-specific T cells, the average number of spots per well for each duplicate was plotted against the number of responder cells per well. Linear regression analysis was used to determine the slope of the line, which represents the frequency of antigen-specific T cells. The assay is considered positive, i.e., reflecting the presence of a primed T cell response, if the binomial probability [62] for the number of spots is significantly different by experimental and control assays, i.e., if the experimental line is statistically significantly different from the control line. To determine differences in *ex vivo* T cell frequencies between groups, Wilcoxon/Kruskal–Wallis analysis was used.

### Online supplemental material.

Ex vivo T cell frequencies and MHC binding assays were performed exactly as described above.

## Supporting Information

Table S1 IC_50_ (nM) of Peptide Binding to HLA (87 KB PDF)

### Accession Numbers

The accession numbers from TubercuList (http://genolist.pasteur.fr/TubercuList/) for Mtb proteins discussed in the manuscript are as follows: 19kd (Rv3763); Ag85B (Rv1886c), CFP10 (Rv3874); ESAT 6 (Rv3875); EsxG (Rv0287), Mtb8.4 (Rv1174c); Mtb9.9A (Rv1793); Mtb39 (Rv1196).

## Abbreviations

APC: antigen-presenting cell
CMV: cytomegalovirus
DC: dendritic cell
LCL: lymphoblastoid cell line
LTBI: latent tuberculosis infection
MHC: major histocompatibility complex
Mtb: *Mycobacterium tuberculosis*
PBMC: peripheral blood mononuclear cell
TB: tuberculosis

## References

[ppat-0030127-b001] (2007). Global tuberculosis control: Surveillance, planning, financing. WHO report 2007.

[ppat-0030127-b002] (2006). The global plan to stop TB, 2006–2015 / Stop TB Partnership.

[ppat-0030127-b003] Flynn JL, Chan J (2001). Immunology of tuberculosis. Annu Rev Immunol.

[ppat-0030127-b004] North RJ, Jung YJ (2004). Immunity to tuberculosis. Annu Rev Immunol.

[ppat-0030127-b005] Oldstone MB (1994). The role of cytotoxic T lymphocytes in infectious disease: History, criteria, and state of the art. Curr Top Microbiol Immunol.

[ppat-0030127-b006] Orme IM (1987). The dynamics of infection following BCG and Mycobacterium tuberculosis challenge in T-cell-deficient mice. Tubercle.

[ppat-0030127-b007] Muller I, Cobbold SP, Waldmann H, Kaufmann SH (1987). Impaired resistance to Mycobacterium tuberculosis infection after selective in vivo depletion of L3T4+ and Lyt-2+ T cells. Infect Immun.

[ppat-0030127-b008] Silva CL, Silva MF, Pietro RC, Lowrie DB (1994). Protection against tuberculosis by passive transfer with T-cell clones recognizing mycobacterial heat-shock protein 65. Immunology.

[ppat-0030127-b009] Flynn JL, Goldstein MM, Triebold KJ, Koller B, Bloom BR (1992). Major histocompatibility complex class I-restricted T cells are required for resistance to Mycobacterium tuberculosis infection. Proc Natl Acad Sci U S A.

[ppat-0030127-b010] Ladel CH, Daugelat S, Kaufmann SH (1995). Immune response to Mycobacterium bovis bacille Calmette Guerin infection in major histocompatibility complex class I- and II-deficient knock-out mice: Contribution of CD4 and CD8 T cells to acquired resistance. Eur J Immunol.

[ppat-0030127-b011] Sousa AO, Mazzaccaro RJ, Russell RG, Lee FK, Turner OC (2000). Relative contributions of distinct MHC class I-dependent cell populations in protection to tuberculosis infection in mice. Proc Natl Acad Sci U S A.

[ppat-0030127-b012] Behar SM, Dascher CC, Grusby MJ, Wang CR, Brenner MB (1999). Susceptibility of mice deficient in CD1D or TAP1 to infection with Mycobacterium tuberculosis. J Exp Med.

[ppat-0030127-b013] Rolph MS, Raupach B, Kobernick HH, Collins HL, Perarnau B (2001). MHC class Ia-restricted T cells partially account for beta2-microglobulin-dependent resistance to Mycobacterium tuberculosis. Eur J Immunol.

[ppat-0030127-b014] Mogues T, Goodrich ME, Ryan L, LaCourse R, North RJ (2001). The relative importance of T cell subsets in immunity and immunopathology of airborne Mycobacterium tuberculosis infection in mice. J Exp Med.

[ppat-0030127-b015] Urdahl KB, Liggitt D, Bevan MJ (2003). CD8+ T cells accumulate in the lungs of Mycobacterium tuberculosis-infected Kb-/-Db-/- mice, but provide minimal protection. J Immunol.

[ppat-0030127-b016] Alderson MR, Bement T, Day CH, Zhu L, Molesh D (2000). Expression cloning of an immunodominant family of Mycobacterium tuberculosis antigens using human CD4(+) T cells. J Exp Med.

[ppat-0030127-b017] Lewinsohn DM, Zhu L, Madison VJ, Dillon DC, Fling SP (2001). Classically restricted human CD8+ T lymphocytes derived from Mycobacterium tuberculosis-infected cells: Definition of antigenic specificity. J Immunol.

[ppat-0030127-b018] Heinzel AS, Grotzke JE, Lines RA, Lewinsohn DA, McNabb AL (2002). HLA-E-dependent presentation of Mtb-derived antigen to human CD8+ T cells. J Exp Med.

[ppat-0030127-b019] Lalvani A, Brookes R, Wilkinson RJ, Malin AS, Pathan AA (1998). Human cytolytic and interferon gamma-secreting CD8+ T lymphocytes specific for Mycobacterium tuberculosis. Proc Natl Acad Sci U S A.

[ppat-0030127-b020] Mohagheghpour N, Gammon D, Kawamura LM, van Vollenhoven A, Benike CJ (1998). CTL response to *Mycobacterium tuberculosis:* Identification of an immunogenic epitope in the 19-kDa lipoprotein. J Immunol.

[ppat-0030127-b021] Lam KS (1997). Application of combinatorial library methods in cancer research and drug discovery. Anticancer Drug Des.

[ppat-0030127-b022] Turner J, Dockrell HM (1996). Stimulation of human peripheral blood mononuclear cells with live Mycobacterium bovis BCG activates cytolytic CD8+ T cells in vitro. Immunology.

[ppat-0030127-b023] Lewinsohn DM, Alderson MR, Briden AL, Riddell SR, Reed SG (1998). Characterization of human CD8+ T cells reactive with Mycobacterium tuberculosis-infected antigen-presenting cells. J Exp Med.

[ppat-0030127-b024] Beckman EM, Melian A, Behar SM, Sieling PA, Chatterjee D (1996). CD1c restricts responses of mycobacteria-specific T cells. Evidence for antigen presentation by a second member of the human CD1 family. J Immunol.

[ppat-0030127-b025] Rosat JP, Grant EP, Beckman EM, Dascher CC, Sieling PA (1999). CD1-restricted microbial lipid antigen-specific recognition found in the CD8+ alpha beta T cell pool. J Immunol.

[ppat-0030127-b026] Moody DB, Ulrichs T, Muhlecker W, Young DC, Gurcha SS (2000). CD1c-mediated T-cell recognition of isoprenoid glycolipids in Mycobacterium tuberculosis infection. Nature.

[ppat-0030127-b027] Yewdell JW, Bennink JR (1999). Immunodominance in major histocompatibility complex class I-restricted T lymphocyte responses. Annu Rev Immunol.

[ppat-0030127-b028] Louise R, Skjot V, Agger EM, Andersen P (2001). Antigen discovery and tuberculosis vaccine development in the post-genomic era. Scand J Infect Dis.

[ppat-0030127-b029] Reed S, Lobet Y (2005). Tuberculosis vaccine development; from mouse to man. Microbes Infect.

[ppat-0030127-b030] Geluk A, van Meijgaarden KE, Franken KL, Drijfhout JW, D'Souza S (2000). Identification of major epitopes of Mycobacterium tuberculosis AG85B that are recognized by HLA-A*0201-restricted CD8+ T cells in HLA- transgenic mice and humans. J Immunol.

[ppat-0030127-b031] Klein MR, Smith SM, Hammond AS, Ogg GS, King AS (2001). HLA-B*35-Restricted CD8 T Cell Epitopes in the Antigen 85 Complex of Mycobacterium tuberculosis. J Infect Dis.

[ppat-0030127-b032] Charo J, Geluk A, Sundback M, Mirzai B, Diehl AD (2001). The identification of a common pathogen-specific HLA class I A*0201-restricted cytotoxic T cell epitope encoded within the heat shock protein 65. Eur J Immunol.

[ppat-0030127-b033] Klein MR, Hammond AS, Smith SM, Jaye A, Lukey PT (2002). HLA-B*35-restricted CD8(+)-T-cell epitope in Mycobacterium tuberculosis Rv2903c. Infect Immun.

[ppat-0030127-b034] Caccamo N, Milano S, Di Sano C, Cigna D, Ivanyi J (2002). Identification of epitopes of Mycobacterium tuberculosis 16-kDa protein recognized by human leukocyte antigen-A*0201 CD8(+) T lymphocytes. J Infect Dis.

[ppat-0030127-b035] Lewinsohn DA, Lines RA, Lewinsohn DM (2002). Human dendritic cells presenting adenovirally expressed antigen elicit Mycobacterium tuberculosis-specific CD8+ T cells. Am J Respir Crit Care Med.

[ppat-0030127-b036] Lewinsohn DM, Briden AL, Reed SG, Grabstein KH, Alderson MR (2000). Mycobacterium tuberculosis-reactive CD8+ T lymphocytes: The relative contribution of classical versus nonclassical HLA restriction. J Immunol.

[ppat-0030127-b037] Wong P, Pamer EG (2003). CD8 T cell responses to infectious pathogens. Annu Rev Immunol.

[ppat-0030127-b038] Klenerman P, Hill A (2005). T cells and viral persistence: Lessons from diverse infections. Nat Immunol.

[ppat-0030127-b039] Kiepiela P, Leslie AJ, Honeyborne I, Ramduth D, Thobakgale C (2004). Dominant influence of HLA-B in mediating the potential co-evolution of HIV and HLA. Nature.

[ppat-0030127-b040] Crubezy E, Ludes B, Poveda JD, Clayton J, Crouau-Roy B (1998). Identification of *Mycobacterium* DNA in an Egyptian Pott's disease of 5,400 years old. C R Acad Sci III.

[ppat-0030127-b041] Salo WL, Aufderheide AC, Buikstra J, Holcomb TA (1994). Identification of Mycobacterium tuberculosis DNA in a pre-Columbian Peruvian mummy. Proc Natl Acad Sci U S A.

[ppat-0030127-b042] Sette A, Fleri W, Peters B, Sathiamurthy M, Bui HH (2005). A roadmap for the immunomics of category A-C pathogens. Immunity.

[ppat-0030127-b043] Peters B, Bui HH, Frankild S, Nielson M, Lundegaard C (2006). A community resource benchmarking predictions of peptide binding to MHC-I molecules. PLoS Comput Biol.

[ppat-0030127-b044] Kamath AB, Woodworth J, Xiong X, Taylor C, Weng Y (2004). Cytolytic CD8+ T cells recognizing CFP10 are recruited to the lung after Mycobacterium tuberculosis infection. J Exp Med.

[ppat-0030127-b045] Sylwester AW, Mitchell BL, Edgar JB, Taormina C, Pelte C (2005). Broadly targeted human cytomegalovirus-specific CD4+ and CD8+ T cells dominate the memory compartments of exposed subjects. J Exp Med.

[ppat-0030127-b046] Noss EH, Pai RK, Sellati TJ, Radolf JD, Belisle J (2001). Toll-like receptor 2-dependent inhibition of macrophage class II MHC expression and antigen processing by 19-kDa lipoprotein of Mycobacterium tuberculosis. J Immunol.

[ppat-0030127-b047] Tobian AA, Potter NS, Ramachandra L, Pai RK, Convery M (2003). Alternate class I MHC antigen processing is inhibited by Toll-like receptor signaling pathogen-associated molecular patterns: Mycobacterium tuberculosis 19-kDa lipoprotein, CpG DNA, and lipopolysaccharide. J Immunol.

[ppat-0030127-b048] Gehring AJ, Rojas RE, Canaday DH, Lakey DL, Harding CV (2003). The Mycobacterium tuberculosis 19-kilodalton lipoprotein inhibits gamma interferon-regulated HLA-DR and Fc gamma R1 on human macrophages through Toll-like receptor 2. Infect Immun.

[ppat-0030127-b049] Fulton SA, Reba SM, Pai RK, Pennini M, Torres M (2004). Inhibition of major histocompatibility complex II expression and antigen processing in murine alveolar macrophages by Mycobacterium bovis BCG and the 19-kilodalton mycobacterial lipoprotein. Infect Immun.

[ppat-0030127-b050] Berthet FX, Rasmussen PB, Rosenkrands I, Andersen P, Gicquel B (1998). A Mycobacterium tuberculosis operon encoding ESAT-6 and a novel low- molecular-mass culture filtrate protein (CFP-10). Microbiology.

[ppat-0030127-b051] Dillon DC, Alderson MR, Day CH, Bement T, Campos-Neto A (2000). Molecular and immunological characterization of Mycobacterium tuberculosis CFP-10, an immunodiagnostic antigen missing in Mycobacterium bovis BCG. J Clin Microbiol.

[ppat-0030127-b052] Sorensen AL, Nagai S, Houen G, Andersen P, Andersen AB (1995). Purification and characterization of a low-molecular-mass T-cell antigen secreted by Mycobacterium tuberculosis. Infect Immun.

[ppat-0030127-b053] Dillon DC, Alderson MR, Day CH, Lewinsohn DM, Coler R (1999). Molecular characterization and human T-cell responses to a member of a novel Mycobacterium tuberculosis mtb39 gene family. Infect Immun.

[ppat-0030127-b054] Coler RN, Skeiky YA, Vedvick T, Bement T, Ovendale P (1998). Molecular cloning and immunologic reactivity of a novel low molecular mass antigen of Mycobacterium tuberculosis. J Immunol.

[ppat-0030127-b055] Gey Van Pittius NC, Gamieldien J, Hide W, Brown GD, Siezen RJ (2001). The ESAT-6 gene cluster of Mycobacterium tuberculosis and other high G+C Gram-positive bacteria. Genome Biol.

[ppat-0030127-b056] Rosenkrands I, King A, Weldingh K, Moniatte M, Moertz E (2000). Towards the proteome of Mycobacterium tuberculosis. Electrophoresis.

[ppat-0030127-b057] Collins ME, Patki A, Wall S, Nolan A, Goodger J (1990). Cloning and characterization of the gene for the ‘19 kDa' antigen of Mycobacterium bovis. J Gen Microbiol.

[ppat-0030127-b058] Borremans M, de Wit L, Volckaert G, Ooms J, de Bruyn J (1989). Cloning, sequence determination, and expression of a 32-kilodalton-protein gene of Mycobacterium tuberculosis. Infect Immun.

[ppat-0030127-b059] Romani N, Gruner S, Brang D, Kampgen E, Lenz A (1994). Proliferating dendritic cell progenitors in human blood. J Exp Med.

[ppat-0030127-b060] Lewinsohn DA, Heinzel AS, Gardner JM, Zhu L, Alderson MR (2003). Mycobacterium tuberculosis-specific CD8+ T cells preferentially recognize heavily infected cells. Am J Respir Crit Care Med.

[ppat-0030127-b061] Sidney J, Southwood S, Oseroff C, Del Guercio MF, Sette A (1999). Unit 18.3: Measurement of MHC/peptide interactions by gel filtration. Current protocols in immunology.

[ppat-0030127-b062] Lewinsohn DM, Tydeman IS, Frieder M, Grotzke JE, Lines RA (2006). High resolution radiographic and fine immunologic definition of TB disease progression in the rhesus macaque. Microbes Infect.

