# Supplementary material for: Immunodominant Tuberculosis CD8 Antigens Preferentially Restricted by HLA-B
Source: PLoS Pathog. 2007 Sep 21;3(9):e127. doi: 10.1371/journal.ppat.0030127 (PMC2323292; doi:10.1371/journal.ppat.0030127)
Supplement: Table S1 — (87 KB PDF) [file ppat.0030127.st001.doc]

Supplemental Table: IC50 (nM) of Peptide Binding to HLA							
Donor	Peptide	HLA Restricting Allele	A*0101	A*0201	A*0202	A*0203	A*0206	A*0301	A*1101	A*2301	A*2402	A*2601	A*3002	B*0702	B*0801	B*1501	B*3501	B*4402	B*4403	B*4501	B*5701	
D466	CFP10 2-9	B4501	29767	25935	48745	 -	 -	 -	 -	 -	 -	 -	 -	 -	 -	12625	 -	287	3869	48	 -	
	CFP10 2-10	B4501	 -	 -	 -	 -	35975	 -	 -	 -	14335	13175		25022	7013	4918		11	131	7.5	 -	
	CFP10 2-11	B4501	28677	7173	727	29	 -	45080	 -	 -	33171	 -	22473	19460	8852	45	 -	38	250	93	 -	
	CFP10 2-12	B4501	1429	29288	9844	369	 -	 -	 -	 -	 -	 -	 -	49713	40359	957	 -	26	167	6.2	 -	
Donor	Peptide	HLA Restricting Allele	A*0101	A*0201	A*0202	A*0203	A*0206	A*0301	A*1101	A*2301	A*2402	A*2601	A*3002	B*0702	B*0801	B*1501	B*3501	B*4402	B*4403	B*4501	B*5701	
D160	CFP10 2-11	B44	28677	7173	727	29	 -	45080	 -	 -	33171	 -	22473	19460	8852	45	 -	38	250	93	 -	
	CFP10 2-12	B44	1429	29288	9844	369	 -	 -	 -	 -	 -	 -	 -	49713	40359	957	 -	26	167	6.2	 -	
	CFP10 2-10	B44	 -	 -	 -	 -	35975	 -	 -	 -	14335	13175		25022	7013	4918		11	131	7.5	 -	
	CFP10 3-11	B44	 -	 -	2377	341	 -	 -	 -	 -	 -	 -	 -	 -	79	796	 -	 -	 -	 -	 -	
	CFP10 85-94	B14	 -	 -	 -	 -	48646	 -	 -	 -	 -	 -	 -	 -	 -	22388	 -	 -	 -	 -	 -	
Donor	Peptide	HLA Restricting Allele	A*0101	A*0201	A*0202	A*0203	A*0206	A*0301	A*1101	A*2301	A*2402	A*2601	A*3002	B*0702	B*0801	B*1501	B*3501	B*4402	B*4403	B*4501	B*5701	
D480	CFP10 3-11	B0801	 -	 -	2377	341	 -	 -	 -	 -	 -	 -	 -	 -	79	796	 -	 -	 -	 -	 -	
	CFP10 3-13	B0801	11314	 -	15362	873	25620	17108	7934	26861	3184	4774		10702	471	382		3043	9374	4097	 -	
	CFP10 2-11	B0801	28677	7173	727	29	 -	45080	 -	 -	33171	 -	22473	19460	8852	45	 -	38	250	93	 -	
	CFP10 2-12	B0801	1429	29288	9844	369	 -	 -	 -	 -	 -	 -	 -	49713	40359	957	 -	26	167	6.2	 -	
	CFP10 3-12	B0801	1268	 -	11791	682	24766	 -	 -	 -	12866	1920		16084	5326	1390		9142	39067	275	 -	
Donor	Peptide	HLA Restricting Allele	A*0101	A*0201	A*0202	A*0203	A*0206	A*0301	A*1101	A*2301	A*2402	A*2601	A*3002	B*0702	B*0801	B*1501	B*3501	B*4402	B*4403	B*4501	B*5701	
D504	Mtb9.8 3-11	A0201	2103	0.19	1.5	0.98	2.7	220	 -	16041	4847	 -	16984	686	8890	299	 -	 -	 -	10566	41084	
	Mtb9.8 2-11	A0201	10870	3.6	1.3	2.0	3.7	5281	9095	1805	11443	72		28202	6732	6134		 -	 -	 -	11143	
	Mtb9.8 1-11	A0201	 -	1160	424	221	44	2302	43378	442	34799	 -		 -	21467	56190		48118	60271	60334	1.1	
	Mtb9.8  3-13	A0201	9.1	116	382	14	4.3	6558	46568	 -	 -	 -		 -	 -	5806		 -	 -	 -	306	
Donor	Peptide	HLA Restricting Allele	A*0101	A*0201	A*0202	A*0203	A*0206	A*0301	A*1101	A*2301	A*2402	A*2601	A*3002	B*0702	B*0801	B*1501	B*3501	B*4402	B*4403	B*4501	B*5701	
D454	Mtb9.8 53-61	B0801	 -	1792	 -	1220	5.7	 -	27723	4105	7799	 -	4071	48	0.22	74	 -	 -	 -	27	 -	
	Mtb8.4 5-15	B1501	42569	5839	10239	19695	4955	125	70	 -	 -	76	2.9	 -	161	10	 -	26868	 -	11157	5757	
	Mtb8.4 5-13	B1501	35090	 -	 -	 -	14773	133	53	 -	10587	59		24162	 -	3.0		12507	7224	68715	11129	
Donor	Peptide	HLA Restricting Allele	A*0101	A*0201	A*0202	A*0203	A*0206	A*0301	A*1101	A*2301	A*2402	A*2601	A*3002	B*0702	B*0801	B*1501	B*3501	B*4402	B*4403	B*4501	B*5701	
D481	CFP10 75-83	B1502	41352	 -	 -	 -	 -	30958	 -	 -	 -	3428	10961	13775	 -	14	 -	1696	14188	406	27206	
Donor	Peptide	HLA Restricting Allele	A*0101	A*0201	A*0202	A*0203	A*0206	A*0301	A*1101	A*2301	A*2402	A*2601	A*3002	B*0702	B*0801	B*1501	B*3501	B*4402	B*4403	B*4501	B*5701	
D432	CFP10 49-58	B3514	28876	 -	 -	 -	 -	 -	 -	2934	1923	3374	 -	8013	 -	410	2011	 -	 -	12548	1372	
	Mtb8.4 32-40	B3514	1150	 -	 -	 -	 -	8550	2518	 -	 -	577	409	 -	6152	64	127	1243	11607	 -	17242	
Donor	Peptide	HLA Restricting Allele	A*0101	A*0201	A*0202	A*0203	A*0206	A*0301	A*1101	A*2301	A*2402	A*2601	A*3002	B*0702	B*0801	B*1501	B*3501	B*4402	B*4403	B*4501	B*5701	
D443	Ag85B 144-153	TBD	 -	 -	 -	34175	 -	2180	33411	 -	 -	570	 -	 -	 -	12624	14568	18788	 -	8483	 -	
	Ag85B 143-153	TBD	 -	3007	 -	 -	27	2498	37864	64971	2594	3837		4939	 -	25921		238	732	15	 -	
HLA peptide binding was determined for peptides representing T cell epitopes.  For each donor, the peptide(s) recognized by T cell clone(s) are shown.  Peptides representing the minimal epitope are shown in bold font.  HLA alleles tested for binding that are relevant to the donor's HLA haplotype are underlined. The IC50 for the peptides for each HLA Class I allele tested is shown.  A dash indicates that the IC50 >50,000 nM.  	
